# Phylogenetic patterns of gene rearrangements in four mitochondrial genomes from the green algal family Hydrodictyaceae (Sphaeropleales, Chlorophyceae)

**DOI:** 10.1186/s12864-015-2056-5

**Published:** 2015-10-21

**Authors:** Audrey A. Farwagi, Karolina Fučíková, Hilary A. McManus

**Affiliations:** Department of Biological Sciences, Le Moyne College, Syracuse, NY USA; Ecology and Evolutionary Biology, University of Connecticut, Storrs, CT USA

**Keywords:** Algae, Chlorophyceae, Hydrodictyaceae, Mitochondrial genome, Sphaeropleales

## Abstract

**Background:**

The variability in gene organization and architecture of green algal mitochondrial genomes is only recently being studied on a finer taxonomic scale. Sequenced mt genomes from the chlorophycean orders Volvocales and Sphaeropleales exhibit considerable variation in size, content, and structure, even among closely related genera. However, sampling of mt genomes on a within-family scale is still poor and the sparsity of information precludes a thorough understanding of genome evolution in the green algae.

**Methods:**

Genomic DNA of representative taxa were sequenced on an Illumina HiSeq2500 to produce 2x100 bp paired reads, and mitochondrial genomes were assembled and annotated using Geneious v.6.1.5. Phylogenetic analysis of 13 protein-coding mitochondrial genes spanning the Sphaeropleales was performed.

**Results:**

This study presents one of the first within-family comparisons of mt genome diversity, and is the first to report complete mt genomes for the family Hydrodictyaceae (order Sphaeropleales). Four complete mt genomes representing three taxa and four phylogenetic groups, *Stauridium tetras*, *Pseudopediastrum boryanum*, and *Pediastrum duplex*, range in size from 37,723 to 53,560 bp. The size variability is primarily due to intergenic region expansion, and intron content is generally low compared with other mt genomes of Sphaeropleales.

**Conclusions:**

Certain gene rearrangements appear to follow a phylogenetic pattern, and with a more thorough taxon sampling genome-level sequence may be useful in resolving systematic conundrums that plague this morphologically diverse family.

**Electronic supplementary material:**

The online version of this article (doi:10.1186/s12864-015-2056-5) contains supplementary material, which is available to authorized users.

## Background

Mitochondrial genomes of Viridiplantae, outside of land plants, have been largely understudied. The disparity among mitochondrial genome data available for different classes of Viridiplantae provides a limited view of the diversity belonging to these groups. Being the closest relatives to land plants, studying the evolution of streptophyte algae has prompted the sequencing of multiple genomes from charophycean lineages [1–5]. Most representatives of mt genomes in the class Chlorophyceae (phylum Chlorophyta, sister group to Streptophyta) come from the order Volvocales, a morphologically diverse clade, ranging from the single celled *Chlamydomonas* to the complex multicellular *Volvox*. Genome size in Volvocales is thought to be correlated with organismal complexity [6], but overall the mt genomes in this order are highly derived, showing great reduction in gene number and considerable diversity in size and non-coding content.

Recently garnering attention, nine mt genomes were sequenced from the sister group of Volvocales, the order Sphaeropleales, previously characterized by only *Acutodesmus obliquus* [7, 8]. Through phylogenetic analyses and examination of gene rearrangement and intron content, variation was seen across the sampling of Sphaeropleales. Considered not as derived as in the Volvocales, the mt genome organization and gene content of *Acutodesmus obliquus* is derived compared to non-chlorophyceans, with a similar general pattern following in the nine other mt genomes surveyed from the order [7, 8]. In this way, *A. obliquus* has been noted as having an intermediate mt genome among the green algal lineages [8]. Volvocalean mt genomes can be highly compact or bloated with repeats, harbor a reduced number of protein coding genes with a broad range of intron densities and GC content, and assemble as both linear and circular, depicting sphaeroplealean mt genomes as comparatively conserved [7, 9, 10].

The known mt genomes of Sphaeropleales consistently share 13 protein coding genes, 4 *rnl* and 2 *rns* fragments, and 22–27 tRNAs. These genomes vary mostly in size and intron content, but it is appropriate to say that the level of mt genome variation is moderate across the order, allowing the genome of *A. obliquus* to generally characterize the order. Though they are sister groups, sphaeroplealean mt genomes are revealing to be substantially different from those in Volvocales in terms of content and variability.

**Fig. 1:**
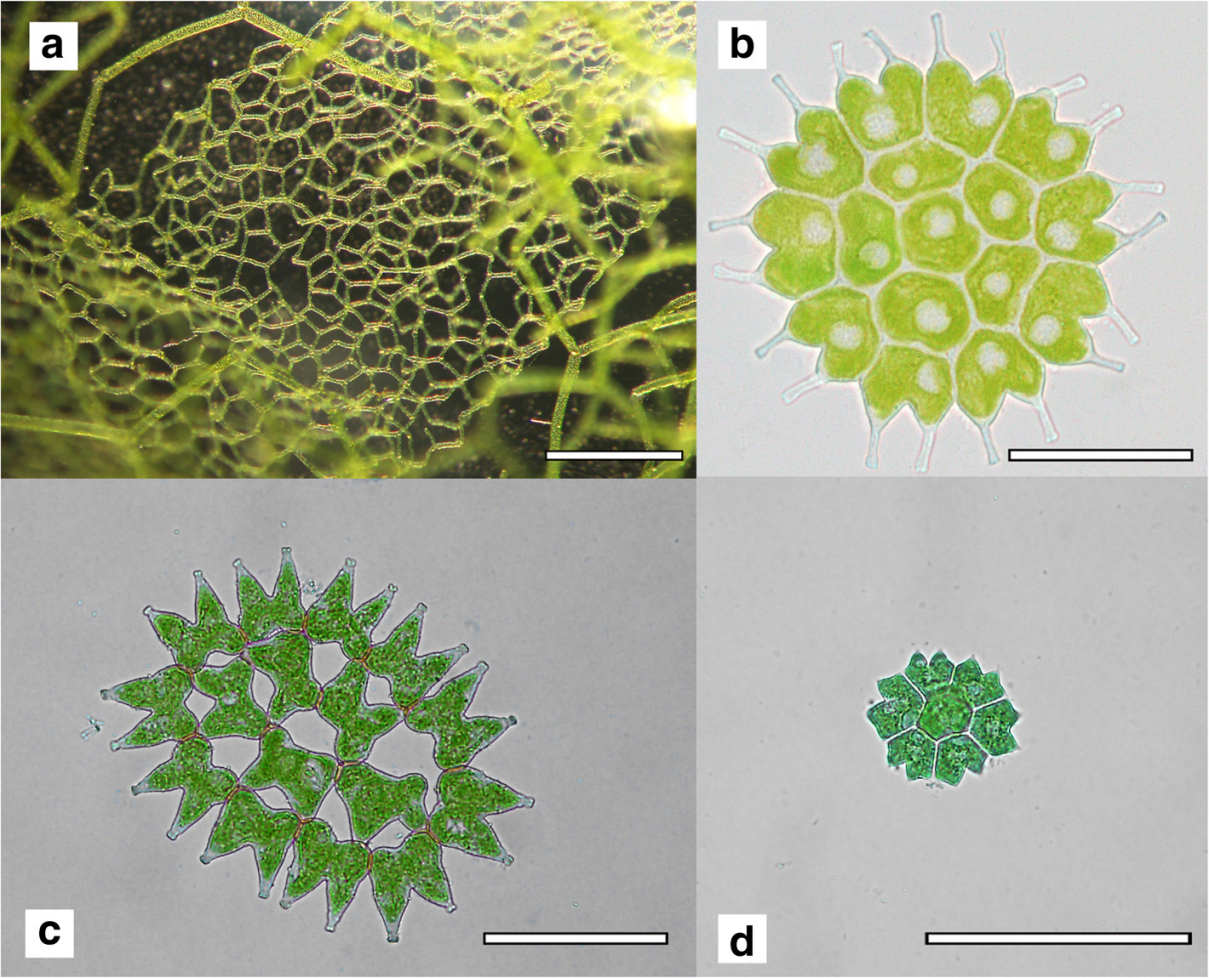
Morphological diversity in the green algal family Hydrodictyaceae. **a** *Hydrodictyon reticulatum,* scale bar = 2 mm, **b** *Pseudopediastrum boryanum,* scale bar = 10 μm, **c** *Pediastrum duplex,* scale bar = 10 μm, **d** *Stauridium tetras*, scale bar = 10 μm

Morphologically, the family Hydrodictyaceae stands out in the order Sphaeropleales due to the characteristic star-like colonies formed by its representative *Pediastrum* Meyen 1829*,* as well as the large, even macroscopic net-like colonies formed by the genus *Hydrodictyon* Roth 1797 (Fig. 1). In spite of the numerous morphological characters available for species delimitation, the systematics of Hydrodictyaceae has been frequently debated and revised over the last decade [11–14], with DNA sequence data revealing extensive cryptic diversity. Several phylogenetic relationships remain to be clarified in the family, particularly the non-monophyly of *Pediastrum duplex* (Fig. 1c) and its relationship to *Hydrodictyon* (Fig. 1a) [13] that represent a taxonomic and evolutionary conundrum. Genome-scale data will likely be necessary to fully understand the molecular and morphological evolution in Hydrodictyaceae.

To contribute to the sampling of mt genomes in Sphaeropleales and test the phylogenetic patterns of gene arrangements and genome architecture within a family, we offer four fully assembled hydrodictyacean mt genomes, as well as protein-coding gene sequences from the fragmentary mt assembly of *Hydrodictyon reticulatum*. As Fučíková et al. [7] only presented fragmentary genome data from Hydrodictyaceae (represented by *Pediastrum duplex*), our study is the first one to report the full mt genomes and is able to investigate the mt genome architecture in this family. We purport that the variability seen thus far across the order is present within a single family, as has been shown for Bracteacoccaceae and Neochloridaceae. These data show promise for resolving phylogenetic relationships among taxa within Hydrodictyaceae while also lending themselves to aid in the further understanding of mt genome evolution across the green algal phylogeny.

## Results

Data collection resulted in 9.8 million reads for *Stauridium tetras* [GenBank:KR026341], 11.1 million reads for *Pseudopediastrum boryanum* [GenBank:KR026342], 8.7 million reads obtained for *Pediastrum duplex* AL0403MN [GenBank:KR026339], and 7.6 million reads for *Pediastrum duplex* PL0501b [GenBank:KR026340]. All four mitochondrial genomes assembled with no gaps, the mean coverage for each was as follows: *P. duplex* AL0403MN 381.3X, *P. duplex* PL0501b 160X, *Ps. boryanum* 86.4X, and *S. tetras* 15.6X. Each genome assembled as a circular molecule (Additional files 1, 2, 3 and 4) and contained 4 *rnl* gene fragments and 2 *rns* fragments, 24 tRNAs (except *Pediastrum duplex* PL0501b with 23 tRNAs), and 13 protein coding genes including *nad1, nad2, nad3, nad4, nad4L, nad5, nad6, cob, cox1, cox2a, cox3, atp6*, and *atp9*. The smallest mt genome belongs to *S. tetras* with 37,723 bp. The mt genome of *Ps. boryanum* has a length of 42,110 bp, and *P. duplex* AL0403MN and *P. duplex* PL0501b mt genomes are 46,416 and 53,560 bp, respectively.

A complete mt genome assembly of *Hydrodictyon reticulatum* was not possible due to contamination and consequently low coverage. Nine of the 13 protein coding genes expected to be present in all Sphaeroplealean mt genomes were sequenced in full and while complete sequences were not recovered for *atp9*, *cox1*, *nad2*, and *nad4L,* the portion of missing data was minimal (1.7 % missing for *Hydrodictyon* and 0.5 % total missing characters in the final trimmed alignment).

**Fig. 2:**
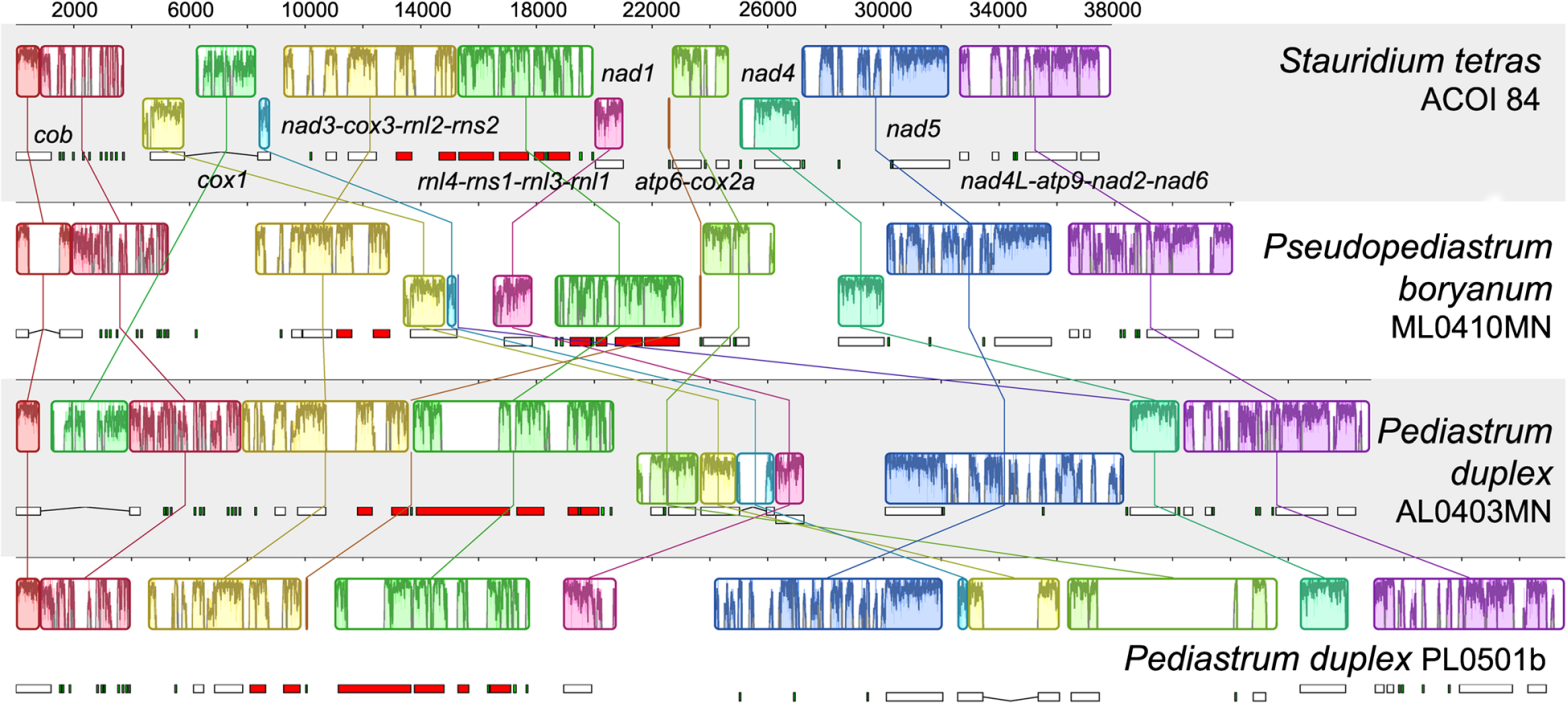
Mauve alignment of four linearized Hydrodictyaceae genomes, beginning at the gene *cob*. Blocks of genes (represented by corresponding colors) are labeled in *Stauridium tetras.* Boxes under each genome map represent protein-coding genes (*white boxes*), rRNA genes (red boxes) and tRNA genes (*small green boxes*)

The position of *nad4L* through trnL-CUA comprises a conserved cluster of 19 genes in all four complete genomes (Fig. 2). The gene cluster trnD-GUC, *nad3, cox3, rnl2, rns2* is also conserved in each genome. *Nad1* has a completely different position in each genome. The *rns* and *rnl* gene fragments are clustered together in each genome, though not in identical orders. Moreover, the order of the fragments is scrambled (i.e., *rnl1*, *rnl2*, *rnl3*, and *rnl4* are not arranged consecutively), but scrambled in the same manner across the genomes (Fig. 2).

**Fig. 3:**
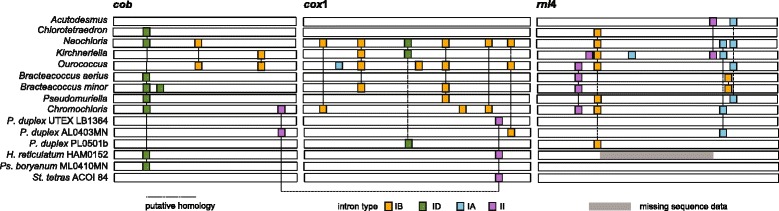
Mitochondrial intron types and distribution in three mitochondrial genes of the order Sphaeropleales

Introns are present only in *cox1*, *cob*, and *rnl4* (Fig. 3). *Stauridium tetras, Hydrodictyon,* and *P. duplex* UTEX LB1364 possess a homologous group II intron in *cox1,* while *P. boryanum* and *P. duplex* AL0403MN contain a group IB intron at a nearby position in *cox1. Pediastrum duplex* PL0501b has a group ID intron roughly in the middle of *cox1*. Introns are present in the *cob* gene in *P. duplex* AL0403MN, *Ps. boryanum*, and *Hydrodictyon* - the latter two are homologous group ID introns, while the first is group II and appears related to the *cox1* group II introns. The *rnl4* gene is uninterrupted in *Ps. boryanum*, *S. tetras*, *P. duplex* UTEX LB1364, and possibly also in *Hydrodictyon* (for which only incomplete *rnl4* data are available). A group IA intron is inserted in the *rnl4* gene of *P. duplex* AL0403MN and a group IB intron is present in *P. duplex* PL0501b, at a different position.

The tRNA specifying threonine is absent from all genomes, and keeping with the deviant genetic code of *Acutodesmus obliquus*, leucine is coded for by UAG (normally a stop codon). The sphaeropleales-specific stop codon UCA is used infrequently in comparison to UAA, but terminates *nad1* in all examined hydrodictyaceans. *Stauridium tetras* is an exception, as this taxon uses UCA in 9 of the 13 protein-coding genes and only uses UAA in *atp6, cox1, cox2a*, and *nad6*.

### Phylogenetic analyses

**Fig. 4:**
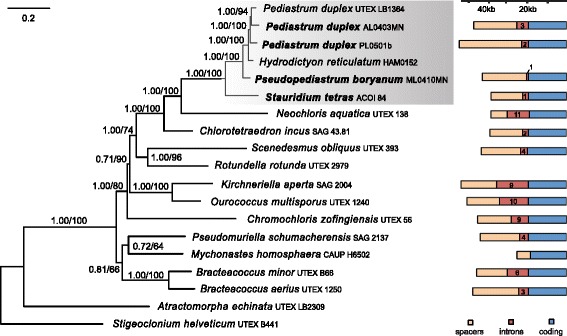
Phylogenetic tree yielded by ML and Bayesian analyses of 13 mitochondrial protein-coding genes. Bayesian consensus tree is shown with support indicated by numbers at branches, representing Bayesian posterior probabilities (BPP) and ML bootstrap (BS) support values, respectively. Scale bar represents the estimat­ed number of substitutions per site. Bars on the right represent mt genome size and composition, as well as total intron number for each taxon

Results of all phylogenetic analyses, including all five ML searches and the Bayesian consensus tree, agreed on the same, completely resolved topology (Fig. 4). All relationships among Sphaeropleales corresponded to the mitochondrial tree presented in Fučíková et al. [7], including *Neochloris* being the closest relative of Hydrodictyaceae and *Chlorotetraedron* being sister to *Neochloris* + Hydrodictyaceae. Relationships among the six included Hydrodictyaceae were well supported, with *Stauridium* being sister to the remaining representatives, *Pseudopediastrum boryanum* sister to *Hydrodictyon* + *Pediastrum duplex. Pediastrum duplex* was recovered as monophyletic with absolute support. Badger analysis of gene rearrangements within Sphaeropleales grouped the four representatives of Hydrodictyaceae as a group with 0.96 support (Additional file 5). *Pseudopediastrum boryanum* + *S. tetras* were resolved as sister to *P. duplex* AL043MN + *P. duplex* PL0501b.

## Discussion

Mitochondrial genomes of Viridiplantae range from hyperinflated and hypervariable in land plants to compact and conserved in some lineages of green algae. From the information available for the class Chlorophyceae, Sphaeropleales appear to be closer to the conservative end of the spectrum, especially compared to their sister order Volvocales [6, 7]. Our study zooms in on the sphaeroplealean family Hydrodictyaceae and compares four fully sequenced and two partially sequenced mt genomes from taxa that span the phylogenetic breadth of the family. This study is the first to report a complete mt genome sequence for the family Hydrodictyaceae, and among the first ones to compare within-family mt genome diversity in green algae.

### Genome size and structure

The large size difference among genomes of different strains is primarily due to expansion of intergenic regions (spacers), and the number of introns does not scale directly with genome size due to high variation in size of individual introns. This correlation appears to be the case across Sphaeropleales: for example, *Neochloris’*s 11 introns (highest intron number in Sphaeropleales) combined are of lesser length than the 9 introns of *Kirchneriella* (second-largest mt genome in Sphaeropleales; Fig. 4). The newly sequenced *Pediastrum duplex* PL0501b has the largest reported mt genome in Sphaeropleales but contains only two introns.

The deepest-diverging hydrodictyacean, *Stauridium tetras*, has a genome size very similar to those of the closest known relatives of Hydrodictyaceae: *Neochloris* and *Chlorotetraedron* (Fig. 4). It is therefore possible that the ancestral mt genome size for the Neochloridaceae + Hydrodictyaceae clade was in the vicinity of 38 kb. However, in contrast to *S. tetras* and *Chlorotetraedron, Neochloris* has a much higher intron content and lower spacer content, which makes such an interpretation of genome size evolution less straightforward as there may be different driving forces behind the proliferation of introns and contraction of intergenic spacers. In the *P. duplex* group the mt genomes appear to have expanded both due to intron acquisition and spacer expansion (especially in *P. duplex* PL0501b).

Within Chlorophyta, the *cox2* gene shows varying degrees of fragmentation and migration. Algae belonging to Prasinophyceae, Ulvophyceae, and Trebouxiophyceae contain orthodox, intact mitochondrial *cox2* genes [15]. Chlorophycean algae exhibit split *cox2* genes, possessing either a mitochondrion-encoded *cox2a* and a *cox2b* fragment that has migrated to the nucleus (*Scenedesmus*-like) or having both *cox2a* and *cox2b* relocated to the nucleus following a reduced-derived pattern (*Chlamydomonas*-like) [15]. As expected, the four newly sequenced genomes presented appear to have a mitochondrial *cox2a* gene, emphasizing the intermediate evolutionary pattern of Sphaeropleales and building a strong case for distinguishing phylogenetic relationships.

Orientating the gene arrangements so that *cob* begins and *nad6* ends the sequence, gene rearrangement is prominent in the regions flanked by the rRNA fragments and “end” of the genomes in both *P. duplex,* while in *S. tetras* and *P. boryanum* gene rearrangement is present only from the “beginning” to the end of the rRNA fragments (Fig. 2). Additional patterns of gene rearrangements are discussed below.

### Intron diversity

Considerable diversity and abundance of introns in the *cob*, *cox1*, and *rnl4* genes was previously described for sphaeroplealean mt genomes [7]. Compared to the diversity found across the order, Hydrodictyaceae have relatively intron-poor mt genomes and, in the strains examined here, no more than one intron per gene is found. Yet, all of the major intron types (groups IA, IB, ID, and II) found across Sphaeropleales are found in Hydrodictyaceae as well (Fig. 3). The group II intron found near the 3′ end of *cox1* in *Hydrodictyon*, *Pediastrum duplex* UTEX LB1364, and *Stauridium* is unique among Sphaeropleales. Interestingly however, BLAST analyses show its relatedness to the *cob* gene of *P. duplex* AL0403MN (71–89 % identity in conserved intron regions including the intronic ORF - a putative reverse transcriptase gene), which appears homologous with an identically positioned *cob* intron in *Chromochloris zofingiensis* (67 % identity in the conserved intron domain V; Fig. 3). A notable 67 % identity in the conserved regions with a *cox1* intron of the red alga *Pyropia* was also detected (however, the intron of *Pyropia* is inserted at a different *cox1* position). The ancestry of this intron is unclear from the available data, but it can be hypothesized that the intron was either present ancestrally in the *cob* gene of all Sphaeropleales and lost many times (even within Hydrodictyaceae), or transferred horizontally into the *cob* gene of *P. duplex* AL0403MN. Either way, this intron may have been present in the ancestral mt genome of Hydrodictyaceae in the *cox1* gene (where it likely arrived from the *cob* gene) and subsequently lost in some members of the family. Much denser taxon sampling will be required to accurately reconstruct the undoubtedly complex evolutionary history of these introns.

The group ID intron found in the *cob* gene of *Hydrodictyon* and *Pseudopediastrum* appears homologous to the same type introns present in several other sphaeroplealeans (especially *Chlorotetraedron* and *Chromochloris* - 68–69 % identity over 50 % or more of the intron’s length), and might therefore be ancestral to the order. In fact, its conserved 3′ end bears significant sequence similarity (78–80 % as determined by BLAST) to identically positioned *cob* introns of *Volvox carteri* and other green algae including *Trebouxia aggregata* (Trebouxiophyceae) and the streptophytes *Chlorokybus atmophyticus*, *Mesostigma viride,* and *Chaetosphaeridium globosum*, as well as several fungi (also similar position in *cob*). The original invasion of this intron into mt genomes might therefore reach deep into the green algal, or even eukaryote, phylogeny.

### Phylogeny of Hydrodictyaceae

Previous studies concerning the systematics of Hydrodictyaceae split *Pediastrum duplex* into two clades, of which “group I” (in our study represented by UTEX LB1364 and AL0403MN) was more closely related to *Hydrodictyon* than to “group II” (here represented by PL0501b). These relationships were mainly supported by the nuclear 28S ribosomal large subunit gene [12, 13] and weakly indicated also by results based on the nuclear ITS-2 and chloroplast *rbc*L, *psa*A, *psa*B genes [13, 16], unpublished data. Contrarily, our study recovered *P. duplex* as a monophyletic species with strong statistical support with *Hydrodictyon* as its sister taxon (Fig. 4). Of course, the present taxon sampling is sparse compared to the studies of nuclear and chloroplast genes, which included dozens of hydrodictyacean isolates. However, it is worth noting that the 28S gene, which supports non-monophyly of *P. duplex*, only contained 94 parsimony-informative characters (less than 5 % of the data set) [13]. The *rbcL* gene is more variable (21.4 % or 253 parsimony-informative sites) [13] but does not provide any statistical support for the grouping of *P. duplex* group I and *Hydrodictyon*. In contrast, our mt data set contains 6473 parsimony-informative sites (60.6 %), and when reduced to reflect the phylogenetic breadth included in McManus and Lewis [13], i.e., Hydrodictyaceae with *Neochloris* as outgroup, 1755 parsimony-informative characters remain (16.4 %).

The pattern of gene rearrangement, along with other characteristics seen in the genomes, is reflected in the phylogeny (Figs. 2 and 4). However, without a much denser sampling of Hydrodictyaceae and their closest relatives, it is impossible to determine the ancestral mt genome structure in the family, with the exception of a handful of conserved blocks of genes. *Stauridium tetras* and *Ps. boryanum*, the two deepest diverging hydrodictyaceans sampled in this study, share the same gene order over a span of 24 genes (from *nad4* to trnL-CUA). This block of genes might be ancestral to Hydrodictyaceae but is not found in closest relatives of the family (*Chlorotetraedron* and *Neochloris*, both of which have mt genomes very differently arranged and are therefore not included in Fig. 2 for simplicity). A cluster of 10 tRNA genes forms a consistent block with the *cob* gene, and this block is likely ancestral to Hydrodictyaceae. The majority of the tRNA gene cluster is also found in *Chlorotetraedron* and *Neochloris* (but not other Sphaeropleales), but it follows *nad1* instead of *cob*. The *nad3-rns2* block and the *rnl4-rnl1* block are in close proximity to each other and on the same strand, with the exception of *Ps. boryanum.* The gene order in both *P. duplex* genomes is identical from *cob* to *rnl1*, and toward the “end” from *nad4* to *nad6*. In *P. duplex* PL0501b, the *rnl4-rnl1* block and the adjacent *cox1* gene appear to have relocated as a single unit relative to AL0403MN. Additional mt genomes from across the family (in particular *P. angulosum*) are necessary to determine if gene order will aid with resolving phylogeny, and especially to reconstruct the relative timing of rearrangement events that shaped the extant genomes.

## Conclusions

Presenting the first mt genomes of the family Hydrodictyaceae, and being one of the only family-level studies of mt genome diversity in the green algae, this study highlights the potential of phylogenetic informativeness and resolving power of fine scale, whole genome studies. Even though this study lacks the taxon sampling to credibly resolve the question of monophyly of *P. duplex*, our data nevertheless show great promise for addressing systematic questions in Hydrodictyaceae. Expanding the representation of mt genome data across the family will further aid in our understanding of mt genome evolution across the green algae.

## Methods

Strains of Hydrodictyaceae were collected and isolated from various fresh bodies of water in the U.S.A. and Australia following procedures from McManus and Lewis [13], or from public culture collections (UTEX, ACOI) (Table 1). Locality information for the strains is included in McManus and Lewis [13]. Cultures were maintained at 20 °C under a 16:8 h light:dark (L:D) cycle on agar slants. The agar slants consisted of a 50:50 mixture of Bold’s basal medium (BBM) [17, 18] and soil water prepared following McManus and Lewis [13].

**Table 1:** Comparison of mitochondrial genomes of Hydrodictyaceae and their closest sphaeroplealean relatives

	*Stauridium tetras*	*Pseudopediastrum boryanum*	*Pediastrum duplex Group I*	*Pediastrum duplex Group II*	*Neochloris aquatica*	*Chlorotetraedron incus*	*Acutodesmus obliquus*
Strain	ACOI 84	ML0410MN	AL0403MN	PL0501b	UTEX 138	SAG 43.81	UTEX 78
GenBank accession No.	**KR026341**	**KR026342**	**KR026339**	**KR026340**	KJ806271	KJ806267	AF204057
Size (bp)	37,723	42,110	46,416	53,560	38,012	38,400	42,919
% GC	32.5	37.8	42.7	45.1	34.4	37.7	36.3
Respiratory protein coding genes	13	13	13	13	13	13	13
Group I introns	0	1	2	2	11	2	2
Group II introns	1	0	1	0	0	0	2
tRNAs	24	24	24	23	24	24	27

Bolded GenBank Accession numbers indicate genomes sequenced for this study

Genomic DNA was extracted from living cells using CTAB extraction or the PowerPlant® Pro DNA Isolation Kit by MoBio Laboratories, Inc. (Carlsbad, CA). DNA was then submitted to Cold Spring Harbor Laboratories for TruSeq library preparation followed by sequencing on Illumina HiSeq2500 to produce 2×100 bp paired reads. *Hydrodictyon reticulatum* was sequenced on a MiSeq instrument at the University of Connecticut set for 250 bp long paired-end reads. Geneious v.6.1.5 (www.geneious.com) [19] was used to pair, trim, assemble, and annotate reads. Contigs of mitochondrial origin were sorted from the paired reads and served as a baseline for the initial map to reference. Assembly was achieved through a series of reference mapping iterations and de novo assembly of contigs until a single, final contig resulted. To verify, paired and trimmed reads were mapped to the final consensus sequence followed by a de novo assembly of the reference reads and reassembly of the contigs produced. Annotations, comprising protein coding genes, large and small ribosomal subunit genes, and tRNAs, were completed for each genome utilizing DOGMA (dogma.ccbb.utexas.edu/) [20], RNAWeasel (http://megasun.bch.umontreal.ca/cgi-bin/RNAweasel/RNAweaselInterface.pl) [21], tRNAscan-SE 1.21 [22], BLAST (http://blast.ncbi.nlm.nih.gov/) [23], Geneious, and previously annotated genomes. Intron type and homology was determined using RNAWeasel and blastn [24, 25]. Preliminary annotations for protein coding genes and large and small ribosomal subunit genes were done with DOGMA and imported into Geneious. After mapping DOGMA annotations to their corresponding genome, open reading frames (ORFs) correlating to *Scenedesmus (Acutodesmus) obliquus* were found using Geneious to verify the start and stop of each gene in conjunction with DOGMA. Annotations from published genomes were used to manually find and annotate rRNA gene fragments in our genomes. Annotations of tRNAs were completed with RNAWeasel and verified using tRNAscan-SE. Gene alignments were created for all protein coding sequences through a translational alignment in Geneious and *rnl* and *rns* fragments were aligned as well (Additional file 6). A Mauve alignment of all four complete hydrodictyaceae mt genomes was produced in which each genome was oriented to begin at *cob* (Fig. 2) [26]. For *Hydrodictyon reticulatum*, protein mt gene sequences were recovered by reference assembly to *P. duplex* (UTEX 1364) gene sequences in Geneious and subsequent reference assemblies to the resulting Hydrodictyon consensus sequences.

### Phylogenetic analyses

Alignment of individual genes and removal of unalignable characters was performed as described in Fučíková et al. [7]. Combined Maximum Likelihood (ML) and Bayesian phylogenetic analyses of the 13 protein-coding mt genes were carried out using the same taxon set, with added hydrodictyacean representatives, and applying the same partitioning scheme and model of evolution (GTR+I+Γ) used in Fučíková et al. [7]. Phycas v.2 [27] was used for Bayesian inference, carrying out 100,000 cycles with polytomies allowed, sampling every 100 cycles. The first 200 trees of the run were discarded as burn-in and Bayesian marginal posterior probabilities (BPP) of splits were estimated from the post-burn-in sample of trees. RAxML [28] was used for a ML search for best tree with five independent searches, and a bootstrapping analysis was carried out for 1000 pseudoreplicates. Badger v.1.02 [29] was used to infer the phylogeny of Sphaeropleales from mt gene order. Badger was run for 5,000,000 generations with otherwise default settings. The data sets supporting the results of this article are included as Additional files 6 and 7.

## Availability of supporting data

All supporting data are included as additional files.

## Additional files

Additional file 1:
**Circular map of mitochondrial genome for** ***Stauridium tetras*** **strain ACOI84, GenBank KR026341.** Drawn by OGDraw and refined in Adobe Illustrator. (PDF 281 kb) Additional file 2:
**Circular map of mitochondrial genome for** ***Pseudopediastrum boryanum*** **strain ML0410MN, GenBank KR026342.** Drawn by OGDraw and refined in Adobe Illustrator. (PDF 289 kb) Additional file 3:
**Circular map of mitochondrial genome for** ***Pediastrum duplex*** **strain AL0403MN, GenBank KR026339.** Drawn by OGDraw and refined in Adobe Illustrator. (PDF 292 kb) Additional file 4:
**Circular map of mitochondrial genome for** ***Pediastrum duplex*** **strain PL0501b, GenBank KR026340.** Drawn by OGDraw and refined in Adobe Illustrator. (PDF 287 kb) Additional file 5:
**Phylogenetic tree resulting from Badger analysis.** (PDF 123 kb) Additional file 6:
**Alignment.** Sequence alignment of individual mitochondrial genes in .nexus file format. (TXT 205 kb) Additional file 7:
**Alignment for Badger, including only genes present in 1 copy and are present in all included taxa (problematic tRNA genes were excluded from the analysis).** (TXT 2 kb)
