# Supplementary figures and images for: Phylogenetic patterns of gene rearrangements in four mitochondrial genomes from the green algal family Hydrodictyaceae (Sphaeropleales, Chlorophyceae)
Source: BMC Genomics. 2015 Oct 21;16:826. doi: 10.1186/s12864-015-2056-5 (PMC4618342; doi:10.1186/s12864-015-2056-5)

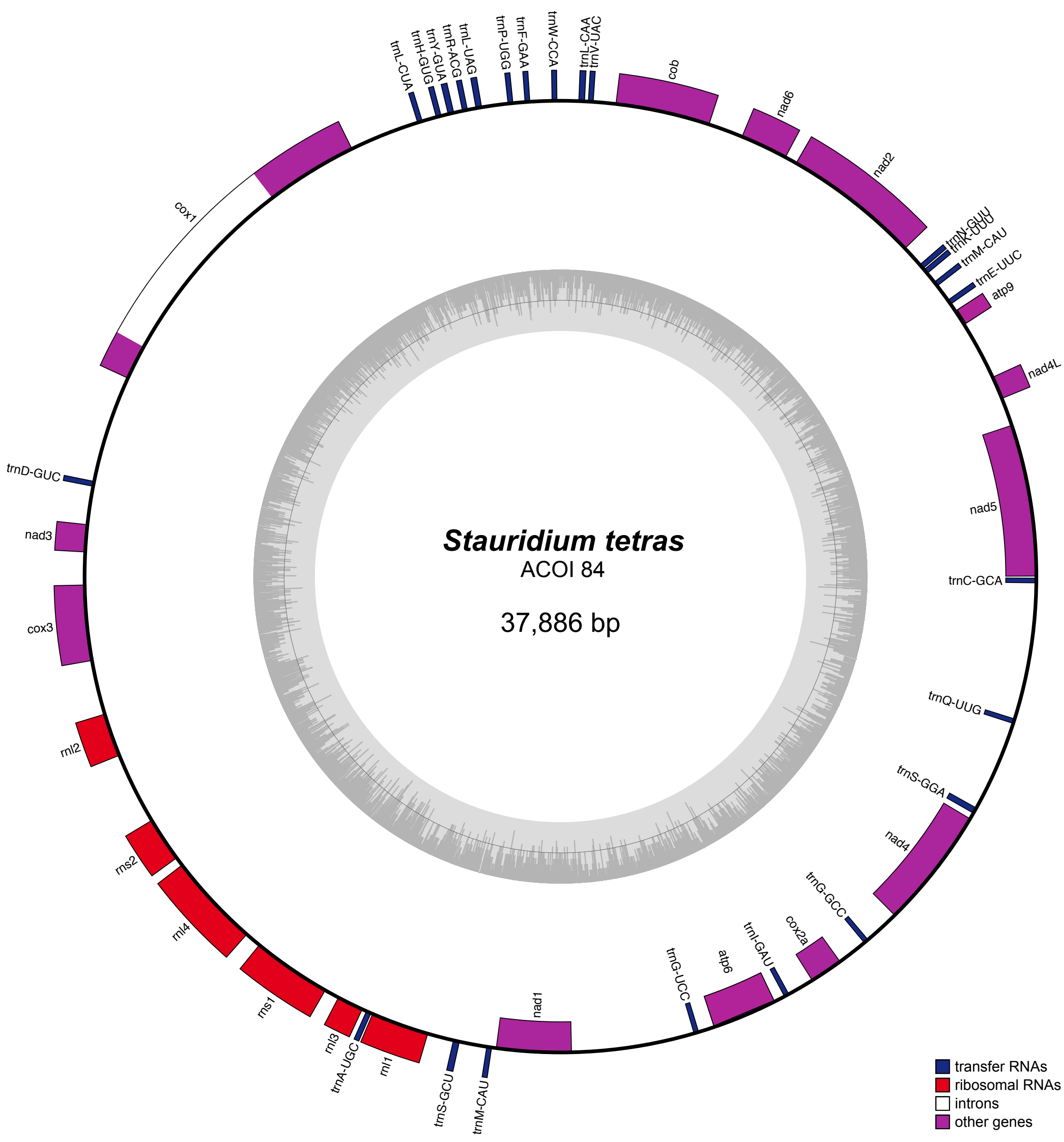

Supplement: Additional file 1: — Circular map of mitochondrial genome for Stauridium tetras strain ACOI84, GenBank KR026341. Drawn by OGDraw and refined in Adobe Illustrator. (PDF 281 kb) [file 12864_2015_2056_MOESM1_ESM.pdf]

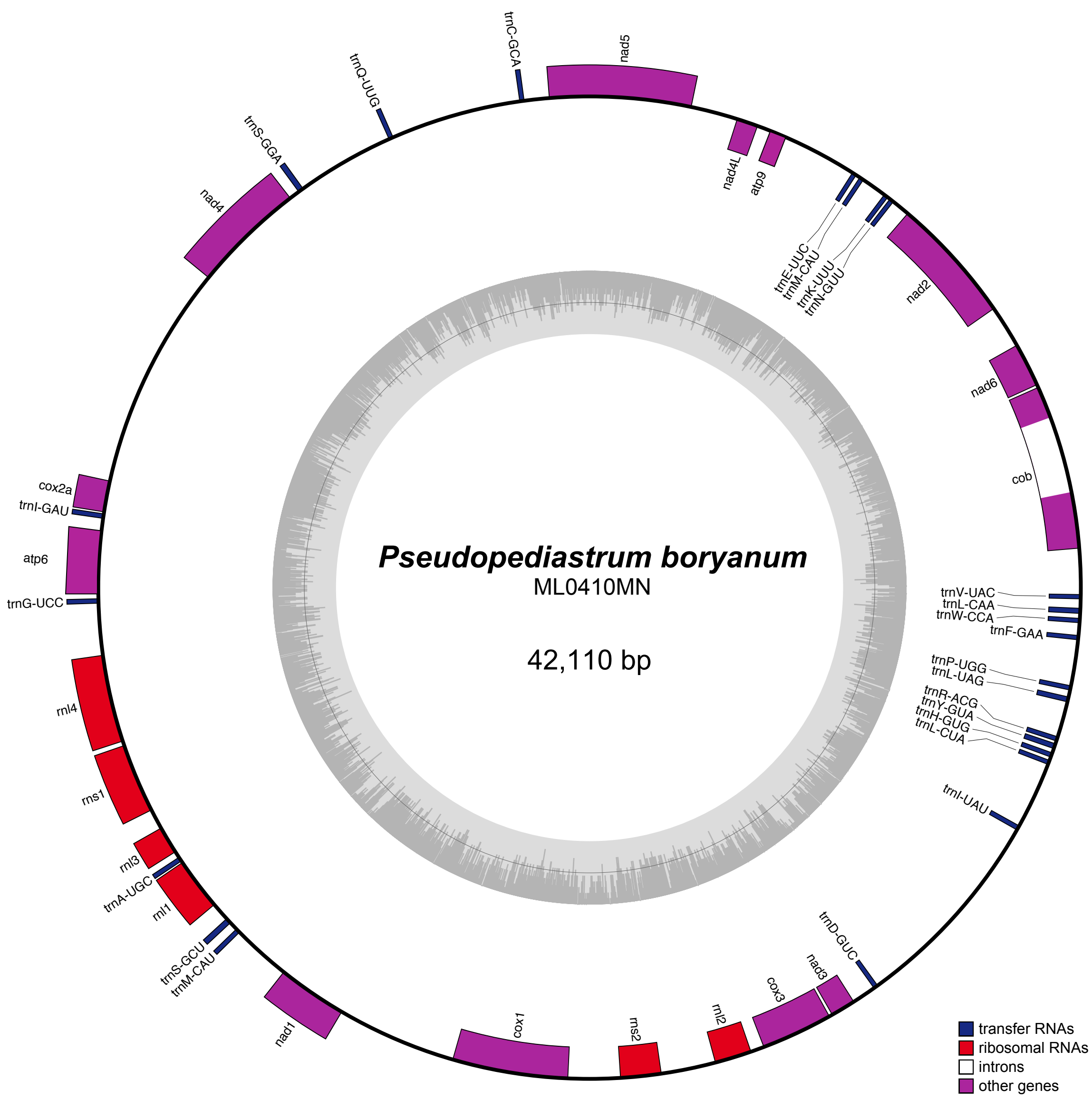

Supplement: Additional file 2: — Circular map of mitochondrial genome for Pseudopediastrum boryanum strain ML0410MN, GenBank KR026342. Drawn by OGDraw and refined in Adobe Illustrator. (PDF 289 kb) [file 12864_2015_2056_MOESM2_ESM.pdf]

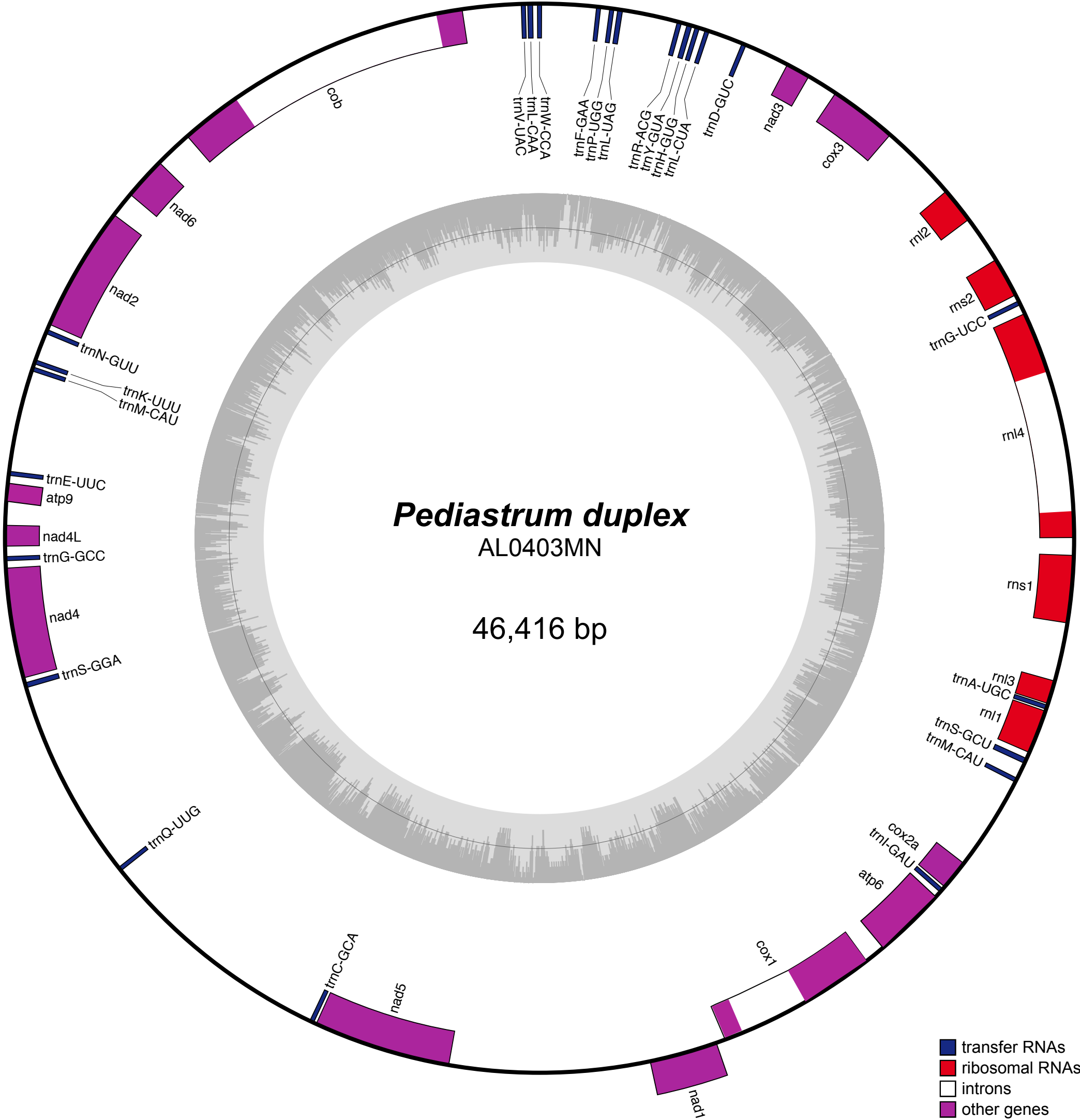

Supplement: Additional file 3: — Circular map of mitochondrial genome for Pediastrum duplex strain AL0403MN, GenBank KR026339. Drawn by OGDraw and refined in Adobe Illustrator. (PDF 292 kb) [file 12864_2015_2056_MOESM3_ESM.pdf]

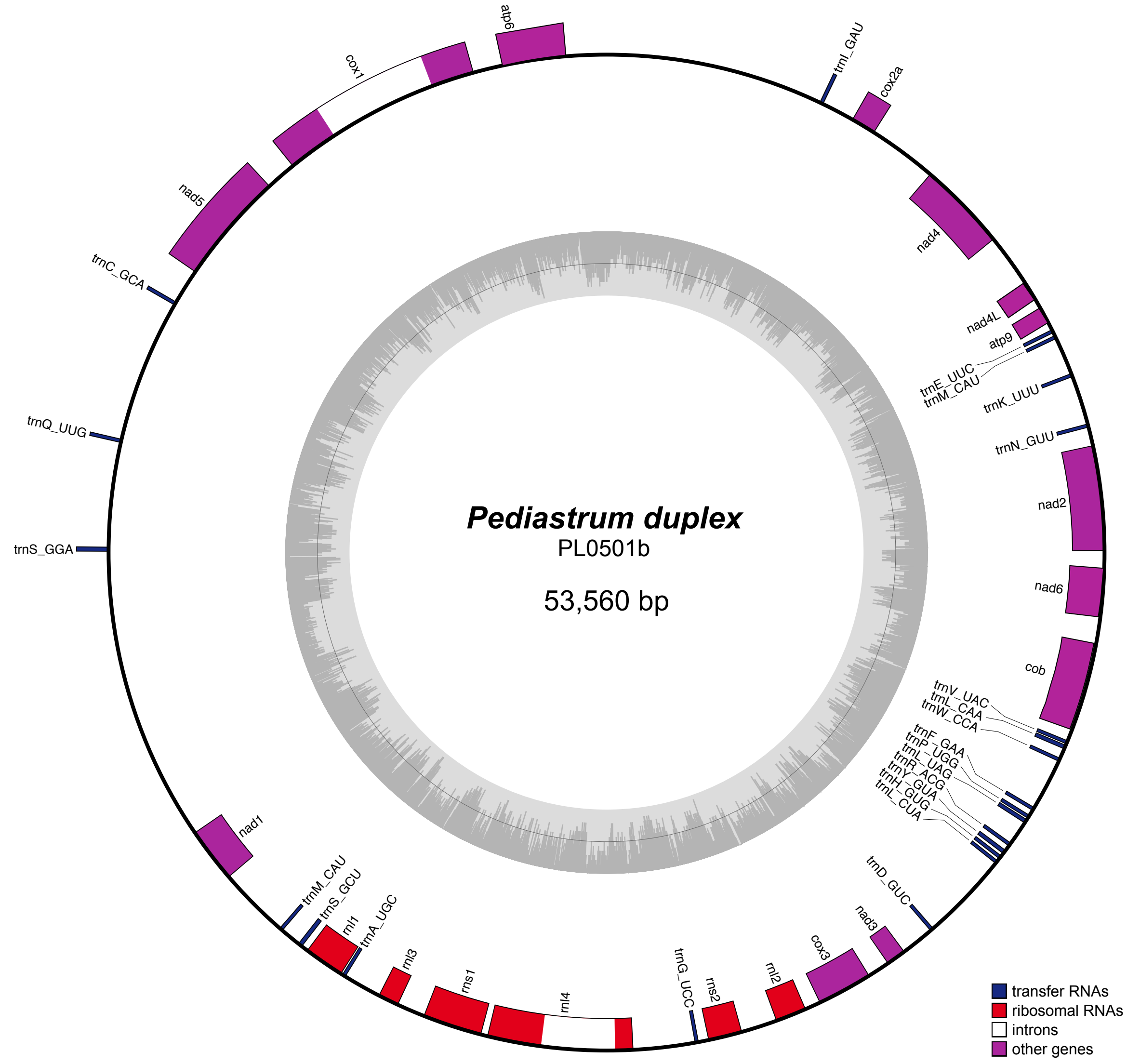

Supplement: Additional file 4: — Circular map of mitochondrial genome for Pediastrum duplex strain PL0501b, GenBank KR026340. Drawn by OGDraw and refined in Adobe Illustrator. (PDF 287 kb) [file 12864_2015_2056_MOESM4_ESM.pdf]

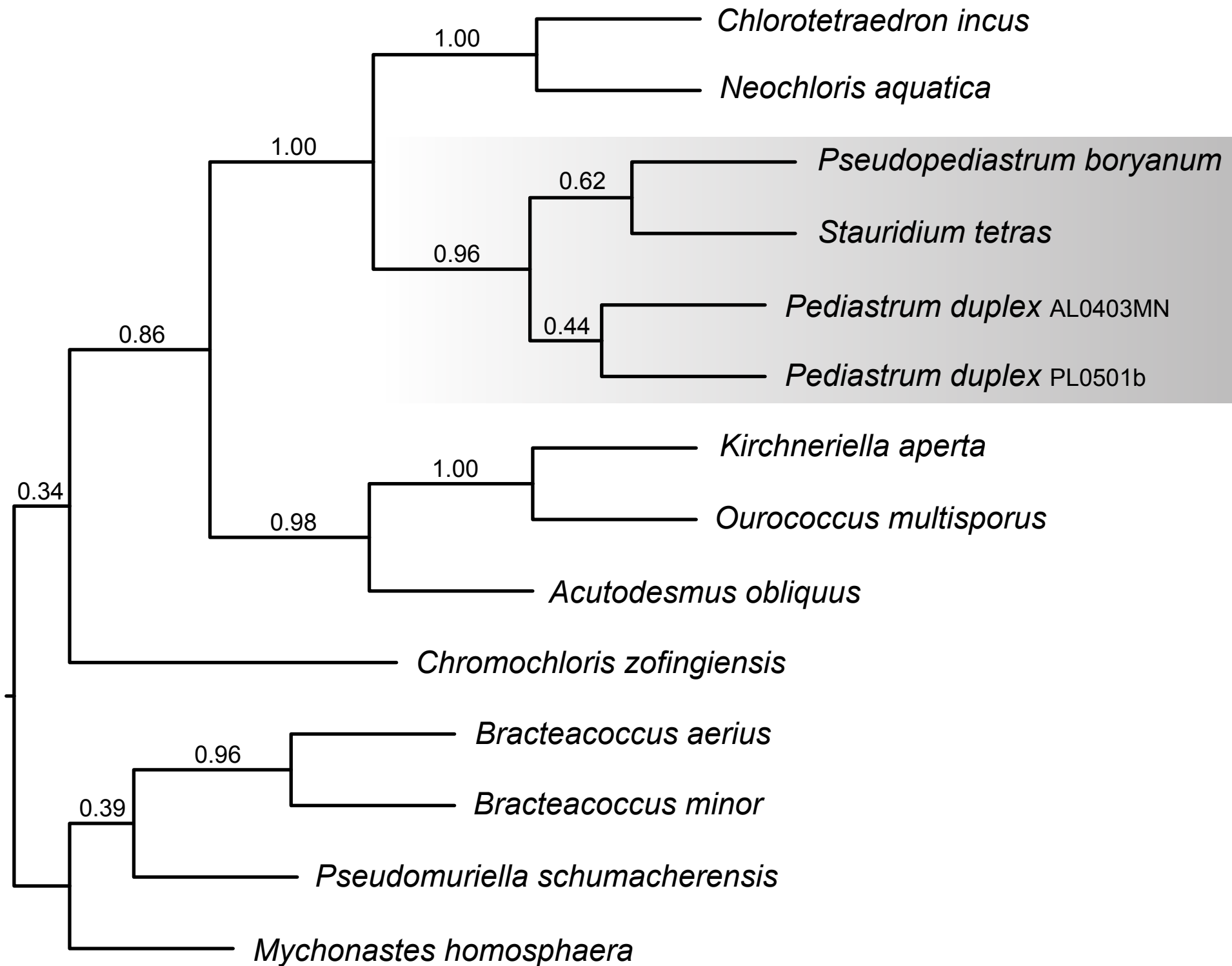

Supplement: Additional file 5: — Phylogenetic tree resulting from Badger analysis. (PDF 123 kb) [file 12864_2015_2056_MOESM5_ESM.pdf]
